# Atrophic Long-Bone Non-Union: Current Insights into Pathogenesis and Management—A Narrative Review

**DOI:** 10.3390/jcm15103611

**Published:** 2026-05-08

**Authors:** Vasileios P. Giannoudis, Helena F. Barber, Vincenzo Giordano, Nikolaos K. Kanakaris, Peter V. Giannoudis

**Affiliations:** 1 Academic Department of Trauma & Orthopaedic Surgery, School of Medicine, University of Leeds, Leeds LS2 9JT, UK; vasileios.giannoudis@nhs.net; 2 Department of Orthopaedic Surgery, Washington University School of Medicine, St. Louis, MO 63110, USA; helena.f.barber@gmail.com; 3 Serviço de Ortopedia e Traumatologia Prof. Nova Monteiro, Hospital Municipal Miguel Couto, Rua Mário Ribeiro 117/2° Andar, Gávea, Rio de Janeiro 22430-160, RJ, Brazil; v_giordano@me.com; 4 Academic Department of Trauma & Orthopaedic Surgery, Leeds Major Trauma Center, Leeds Teaching Hospitals NHS Trust, Leeds LS1 3EX, UK; n.kanakaris@nhs.net; 5 NIHR Leeds Biomedical Research Center, Chapel Allerton Hospital, Leeds LS7 4SA, UK

**Keywords:** non-union, mesenchymal stem cells, bone healing

## Abstract

One of the complications of both surgical and non-surgical treatment of fractures is the development of non-union. The 5–10% incidence of non-union quoted in the international literature is thought to be an underestimate of the real magnitude of this clinical problem. The etiology of atrophic non-union is multifactorial, involving biological, mechanical, infectious, and host-related factors. Much of the evidence regarding its pathogenesis is heterogenous and largely hypothesis generating. This heterogenicity has contributed to the wide range of treatment strategies used to address an atrophic non-union, with variable success rates. This structured narrative review summarizes current insights into the pathogenesis of atrophic non-union, including the inflammatory and immune response, the role of mesenchymal stem cells, bone morphogenetic protein, and the mechanisms of remodeling and angiogenesis. It also outlines an algorithmic approach to management, including the exclusion of occult infection, assessment of mechanical stability, optimization of modifiable host factors, and, finally, a graded approach to enhance the biological and mechanical environment of the non-union.

## 1. Introduction

The Food and Drug Administration defines fracture non-union as the failure of a fracture to progress to osseous healing within nine months following injury, in the absence of progressive signs of healing on serial radiographs over three consecutive months [1]. In the United States, over 100,000 fractures per year are estimated to be non-union [2]. In a recent epidemiological study in Scotland, an overall incidence of non-union of 18.94 per 100,000 population per annum was reported [3].

Non-union is classified as septic or aseptic according to the presence or absence of bacteria, respectively, at the fracture site. The radiographic appearance of the non-union is used to establish whether it is hypertrophic or atrophic: hypertrophic non-union results from insufficient fracture stabilization and is associated with extensive callus formation; in atrophic non-union, fracture stabilization is usually adequate, but there may be dysfunction of the local biological activity, which leads to little, or no, callus formation.

**Figure 1:**
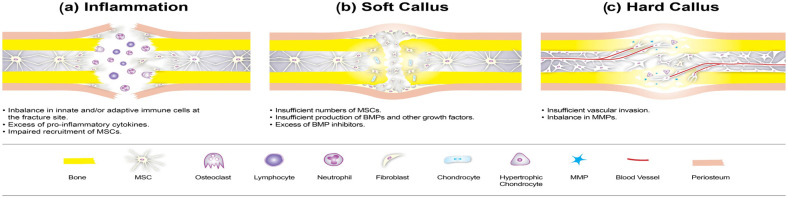
The three stages of indirect fracture repair and the cell types involved: (**a**) Inflammatory stage, characterized by the formation of hematoma, the influx of inflammatory cells, and the recruitment of MSCs. (**b**) The chondrogenic, or “soft callus”, stage, characterized by the maturation of granulation tissue into cartilaginous callus. (**c**) The osteogenic, or remodeling, stage, which encompasses revascularization, mineralization, and bone remodeling.

The indirect fracture repair process, which is characterized by callus formation comprises three stages: the inflammatory, chondrogenic, and osteogenic stages [4] (Figure 1). The hallmark of the inflammatory stage is the formation of fibrin clot/hematoma, the influx of inflammatory cells, the recruitment of repair cell precursors (mesenchymal stem cells, MSCs), and, finally, the formation of a granulation tissue comprising fibroblasts and endothelial cells (Figure 1a). During the chondrogenic, or “soft callus”, stage, granulation tissue matures into cartilaginous callus; however, concomitant formation of periosteal bony callus and osteoclast-driven resorption of dead bone ends occurs on the fracture periphery (Figure 1b). The concluding osteogenic, or “remodeling”, stage in which there is remodeling of soft callus into a hard bony callus includes revascularization, mineralization, cartilage tissue resorption, and longer-term bone remodeling (Figure 1c). Numerous distinct cell types play a critical part in these finely tuned and orchestrated processes: maturation of the cells into intermediate cell phenotypes and production of chemokines and growth factors enable the influx, maturation, and differentiation of other cells.

The etiology of non-union is thought to be multifactorial and linked to patient-related factors, and mechanical and biological issues [5]. While hypertrophic non-unions are widely accepted to be due to inadequate mechanical stability, the pathogenesis of atrophic non-unions remains poorly understood. Most theories explaining the biological causes of atrophic non-union pertain to the systemic or local deficiency in cells and/or cytokines at the fracture site. However, inadequate mechanical stability, host-related factors, and occult infection may also hinder callus formation and contribute to the development of non-union. Radiographs may distinguish between hypertrophic and atrophic non-union, but they cannot accurately differentiate septic from aseptic non-union or reliably identify cases with inadequate mechanical environment and inappropriate strain across the fracture site.

The aim of this study was to investigate the latest mechanisms implicated in the etiopathogenesis of atrophic fracture non-union and recent trends in regard to its management.

## 2. Methodology

### 2.1. Objective

This article presents a narrative review of the latest theories that have been suggested in reference to the etiopathogenesis of long-bone atrophic non-union. We aim to synthesize theories on this complex issue and suggest practical options for the management of atrophic non-union.

### 2.2. Literature Search and Study Selection

A literature review was conducted using PubMed, Embase, Scopus, and the Cochrane Database of Systematic Reviews. This search incorporated combinations of the following keywords: “atrophic nonunion”, “pathogenesis”, “etiology”, mesenchymal stem cells”, “bone morphogenetic proteins”, “osteoimmunology”, “inflammation”, “immune response”, “angiogenesis”, “management”, and “diamond concept”. A representative PubMed search strategy included ((“atrophy”[MeSH Terms] OR “atrophy”[All Fields] OR “atrophic”[All Fields]) AND (“nonunion”[All Fields] OR “nonunions”[All Fields]) AND (“etiology”[MeSH Subheading] OR “etiology”[All Fields] OR “pathogenesis”[All Fields])) AND (2005:2026[pdat]). This was repeated with combinations of the above keywords, while retaining “atrophic nonunion” in the search strategy. The last search was conducted on 19 April 2026, and it included articles from 2005 to 2026. Studies were included if they investigated the pathogenesis of atrophic non-union and reported on the etiology or management of atrophic non-union. Studies encompassed experimental studies in both human and animal subjects, observational studies, and relevant review articles that were published in peer-reviewed journals and were available in English. Studies were excluded if they focused only on hypertrophic non-union, or fracture healing in the absence of a discussion on non-union; lacked sufficient detail on the pathogenesis or management of atrophic non-union; or were not available in English. Articles were prioritized based on their relevance to the pathogenesis or management of atrophic non-union, with an initial emphasis on studies published since 2005. Additional studies were identified via citation tracking of key studies, as well as iterative, focused searches to further refine and expand upon each proposed theory and management options. Key studies were defined as those with a clear focus on the etiology of atrophic non-union, particularly those involving human, or those frequently cited in the literature. These additional searches identified landmark papers published prior to 2005 that were included given their relevance and influence on additional research into atrophic non-unions. As this was a narrative review, no formal risk-of-bias assessment of the included studies was performed.

## 3. Theories on Pathogenesis of Atrophic Long-Bone Non-Union

Overall, three mechanisms have dominated the current discussion of the biologic etiopathogenesis of atrophic non-union.

### 3.1. Uncontrolled Behavior of Immune Cells

The first theory relates to an uncontrolled behavior of immune cells. During the inflammatory stage of fracture repair, platelet aggregation and clot formation lead to a massive release of platelet-derived chemokines and growth factors that attract other immune and non-immune cells to the damaged area. Neutrophils enter this area first, followed by macrophages and adaptive immune cells, such as T- and B-lymphocytes. The influx of immune cells is associated with increased amounts of interferon-g, tumor necrosis factor- α (TNF-α), and other pro-inflammatory cytokines at the fracture site. Changes in the local cytokine milieu lead to the activation of monocyte-derived osteoclast progenitors and their maturation to functional osteoclasts that are able to resorb dead bone ends. According to one theory, an individual’s adaptive immune profile in the circulation and the delayed termination of inflammation have a strong bearing on endogenous bone regeneration [6,7,8]. Excess tumor necrosis factor-α, in particular, has an adverse effect on the survival, proliferation, and differentiation of MSCs that also accumulate at the fracture site in a similar time frame (Figure 1a).

Recent studies focusing on the uncontrolled behavior of immune cells investigate the role of osteoimmunology in fracture healing and subsequent non-union [9,10,11]. The fracture hematoma microenvironment, cytokine, and growth factor content organize the initial inflammatory response and subsequent immune response required for fracture healing [12,13]. Fracture healing is known to be delayed in immunosuppressed populations, with differences seen as early as excessive pro-inflammatory cytokines in the fracture hematoma both in animal models and in immunologically restricted patients [14,15,16,17]. While an initial local inflammatory response is critical to fracture healing, dysregulated, prolonged, or excessive inflammation can be detrimental and contribute to non-union.

In addition, other studies have elucidated the impact of immune dysregulation in atrophic non-union. Animal models of atrophic non-union have demonstrated an aberrant inflammatory reaction marked by increased expression of inflammatory cytokines, including TNF-α, IL-1β, MMP-9, and -13 [18,19], while other studies report elevated serum biomarkers indicative of a prolonged inflammatory state in atrophic non-union [18,20]. Unwarranted or prolonged inflammation, such as in the case of overexpression of TNF-α, leads to the overactivation of osteoclasts relative to osteoblasts and subsequent bone resorption or erosion [21,22,23,24]. Wagner et al. demonstrated significantly increased expression of RANKL and enhanced osteoclast activity across all time points in an atrophic non-union animal model. In a follow-up study, they regulated TNF-α with etanercept and found restoration of bone regeneration [19,23]. The presence of osteoclasts and their increased activity have been confirmed in human non-union tissue in other studies, including one by Schira et al., who noted increased expression of RANKL and active osteoclasts in well-established human scaphoid non-union when compared to surrounding cancellous bone [25,26,27,28].

The complex balance and interactions of multiple inflammatory mediators and subsequent osteogenic response are crucial for successful fracture healing. The process underlying the immune behavior in atrophic non-union should not be considered a dormant process, but one of dysregulated biological activity. The exact interactions and optimal homeostasis continue to be defined to better guide treatment interventions for atrophic non-union.

### 3.2. Local or Systemic Deficiency in MSCs

The second theory postulates that a local or systemic deficiency in mesenchymal stem cells (MSCs) or bone morphogenetic protein (BMP) expression drives atrophic non-union. MSCs are progenitors of chondrocytes and osteoblasts and are critical for the formation of cartilaginous and periosteal calluses. MSCs are recruited locally into the fracture site from adjacent bone marrow and periosteum [29] (Figure 1b). Chemotaxis of MSCs is mediated via platelet-derived growth factors (PDGFs), stromal-derived factor-1 (SDF-1), and other strong chemokine molecules that are released upon platelet activation. The systemic recruitment of MSCs from a distant site has been demonstrated in animal models; however, there is no conclusive evidence of such recruitment in humans.

Hernigou and Beaujean were the first to report reduced levels of osteoprogenitor/MSC at non-union sites and proposed a new method of treating fracture non-union by injecting autologous bone marrow MSCs into non-union sites [30]. The presence of MSCs in human fracture hematomas has since been proven by a rigorous phenotypic and functional analysis [31,32], and MSCs in late, atrophic non-union human tissues have been confirmed to be functionally inferior to gold-standard bone marrow MSCs [25,33,34]. While uncultured MSCs exhibit significantly lower growth factor expression and increased signs of quiescence, animal and human studies have both demonstrated that they often retain osteogenic activity in vitro and can be induced to differentiate under more favorable conditions [33,34,35,36,37,38,39]. It remains unclear whether deficiency of MSCs in non-union sites is a consequence of their reduced recruitment at the early stages of repair (Figure 1a) or the result of the non-union process itself.

A potential deficiency in the production of bone morphogenetic proteins (BMPs) or an excessive production of their antagonists, such as Noggin, by non-immune cells located in the granulation tissue, in the periosteum, or within the soft regenerative callus itself, has long been recognized as another potential cause of non-union in animal models [40]. There is a considerable amount of literature investigating BMP and BMP inhibitor profiles during fracture repair in both humans and animal models; however, the results remain inconclusive, primarily because of large redundancy in the signaling pathways involved. The assessment of relative contributions of individual BMP molecules and their antagonists, therefore, is rather difficult. In terms of transferring this knowledge to future treatment strategies, some authors have suggested that blocking BMP antagonists could be more effective than adding exogenous BMPs in reducing the incidence of non-union [41].

In looking at the second theory, an inherent MSC deficiency at the bone marrow level in patients with non-union has also been considered. A reduction in bone marrow MSCs (distant to the fracture site) has been documented in non-union patients compared with controls and acute trauma patients [33,42,43,44]. Again, this may be a consequence, rather than the cause, of non-union. Further work is needed to study PDGF and SDF-1 chemokine responsiveness of MSCs in patients who healed successfully compared with those who developed non-union. Such a prospective study would address whether a defect in MSC migration is responsible for their reduced numbers in patients with non-union.

Despite the ambiguity in defining a precise point of MSC or BMP dysregulation in fracture healing, both MSCs and BMPs are often used in the management of atrophic non-union with considerable patient success [45,46,47,48,49]. In particular, BMP-2 and BMP-7 have shown to play major roles in promoting fracture healing in both human studies and animal models [47,48,49,50,51,52,53,54,55,56,57,58]. Their therapeutic success only further supports the critical role of MSCs and BMPs in the pathogenesis of atrophic non-union.

### 3.3. Cells Acting at Late Stages of Fracture Repair

The third and final theory implicates dysfunctional behavior of cells acting at late stages of fracture repair. Cells that act at late stages of fracture repair include differentiating chondroblasts and endothelial cells that are involved in the remodeling of soft callus into hard callus tissue (Figure 1c). Matrix metalloproteinases (MMPs) and their tissue inhibitors mediate the transition of extracellular matrix from type II to type I collagen and are vital during the remodeling stage. Preclinical studies and in vitro data suggest that alterations in immune cell profiles and/or BMP profiles may disrupt MMP activity and contribute to delayed or failed healing [59,60] (Figure 1c). However, direct causal relationships in human atrophic non-union remain incompletely defined.

Additionally, there may be damage to the angiogenic response and revascularization of the fracture site, which is characterized by complex interactions between vascular endothelial cells/progenitors and MSC descendants, such as chondroblasts and osteoblasts. The role of impaired vascularization in the development of atrophic non-union continues to be a subject of debate [61]. More recent hypotheses, largely supported by preclinical models, focus on the temporal importance of VEGF and angiogenesis in fracture healing, as well as the balance of the expression of angiogenic and osteogenic proteins, such as BMP-2 and BMP-4 [62,63].

While known to be important in regulating the fate of MSCs and bone remodeling and repair, there are limited studies investigating the role of MMP dysregulation specific to atrophic non-union in humans. Recent studies have begun to investigate the effect of the CtBP1/2 complex on inhibition of MMPs and subsequent risk of non-union: however, these findings are primarily derived from animal and mechanistic studies [64,65,66,67]. MMP-9 and -13 have been noted by multiple studies as important regulators of bone healing. Knock-out models of MMP-9 and MMP-13 show delayed bone healing, supporting a functional role in fracture repair in animal models, while more recent genetic studies in both preclinical and human studies have found polymorphisms of MMP-13 to be protective against non-union [68,69,70,71]. On the contrary, a significant upregulation of MMP-9 and -13 has been seen in multiple studies of atrophic non-union; however these findings are largely descriptive and do not clarify whether this represents a causative or compensatory mechanism [19,27]. Similar to the BMP pathway, the complex interactions and functional redundancy among MMPs make it challenging to isolate specific changes and define their precise roles in atrophic non-unions [72,73].

Impaired vascularity is often proposed as a causative factor of atrophic non-union, with multiple studies demonstrating that disruption of vascular supply or inhibition of angiogenesis can induce atrophic non-union in animal models [74,75,76,77,78]. Further supporting this theory is the ability to promote fracture healing and osteogenesis with the addition of vascular endothelial growth factor (VEGF), as demonstrated in preclinical studies [79,80,81]. While inadequate vascularization will lead to non-union development, multiple human studies with histological evaluation of established non-union demonstrate adequate vascularization when compared to healing fractures or hypertrophic non-union [25,26,27,62,82,83,84,85].

Garcia et al. found no difference in vessel density at any time point comparing atrophic non-union and united fractures; however, other studies found that atrophic non-union had a lower vessel density in early fracture healing, followed by a recovery to normal or even elevated levels in an established non-union [62,75,83,86]. Similarly, VEGF has been found to be at equal levels, or even overexpressed in non-union compared to controls, suggesting active angiogenesis [43,87]. Discussion continues as to whether the upregulation of VEGF and increased vessel density are a causative or compensatory factor of non-union. Vascularity is essential for fracture union. Although the precise dysregulation of the angiogenesis pathway that contributes to atrophic non-union is not fully understood, it is likely a multifactorial process that depends on appropriate timing and balance of angiogenic factors alongside other key players in the osteogenic cascade.

## 4. Management of Atrophic Long-Bone Non-Union

### 4.1. The Impact of Atrophic Long-Bone Non-Union

Treatment of atrophic long-bone non-union has been shown to have a high financial impact, with average direct costs reported as CN $11,800, US $11,333, and £ 29,204 [88,89]. The key driver for the overall cost, however, is the indirect costs secondary to productivity losses. Strategies that focus on addressing the underlying cause of atrophic non-union to achieve successful faster healing, leading to early restoration of function and resumption of work, would optimize outcomes in and reduce the financial burden on patients with non-union.

### 4.2. Treatment Options for Atrophic Long-Bone Non-Union

Different treatment modalities have been suggested for aseptic long-bone atrophic non-unions, ranging from minimally invasive methods of injection of bone marrow aspirate, growth factors, delivery of bone grafts, and dynamization of the fracture environment to more interventional procedures, consisting of revision of fixation with additional bone grafting [90,91,92,93,94]. In addition, non-invasive stimulation procedures have been used, such as low-pulse ultrasound treatment and electromagnetic stimulation, with positive results [90,95,96,97,98]. The choice of treatment depends on the size of the non-union gap, the biological potential of the non-union environment, and the state of the mechanical environment, amongst others.

### 4.3. Non-Operative Management of Atrophic Long-Bone Non-Union

Non-operative management of atrophic long-bone non-union includes modalities such as low-intensity pulsed ultrasound (LIPUS) and electromagnetic stimulation. When used in the appropriate patient population and clinical scenario, these options have been found to have high rates of successful union.

LIPUS has been approved for the treatment of non-union in the Unites States since 2000 and offers a non-invasive method of biophysical stimulation of the fracture site to promote union [99]. The utility in the management of strophic non-union remains debated. Although hypertrophic non-unions appear to have a greater benefit from LIPUS than atrophic non-unions, LIPUS remains a viable treatment option for atrophic non-unions, with reported fracture healing rates ranging from 76.9 to 93% [96,100,101]. A recent review identified several key factors necessary for successful LIPUS therapy, including appropriate fracture site stability, an interfragmentary gap < 10 mm, accurate placement of the transducer, appropriate treatment duration, and high daily compliance [99]. The standard LIPUS treatment protocol involved daily self-administered sessions—typically lasting 20 min—with the total number of treatments increasing in proportion to the duration of non-union [99].

Pulsed electromagnetic field (PEMF) stimulation is another non-invasive biophysical technique used to promote fracture healing. Treatment protocols vary but typically involve wearing external electrodes for several hours per day [102,103,104,105]. Similar to LIPUS, its role in the management of atrophic non-union remains an area of ongoing investigation. While systematic reviews demonstrate a trend toward clinical benefit, they also highlight a lack of high-quality evidence supporting superiority over control groups [97,103]. Contraindications to PEMF therapy often include active infection, inadequate mechanical stability, and a fracture gap greater than 5–10 mm [104,105]. PEMF is also contraindicated in patients with certain cardiovascular risks, including those with a unipolar pacemaker [106]. Reported union rates for non-union treatment range from 75% to 90%; however, many studies do not distinguish between atrophic and hypertrophic non-unions [104,105,107].

As with LIPUS, these non-operative modalities are not appropriate as standalone treatments for atrophic non-unions in the setting of inadequate mechanical stability or large fracture gaps. However, they represent low-risk, non-invasive options that may be considered in high-risk surgical patients with favorable non-union characteristics.

### 4.4. The “Diamond Concept” for Surgical Management of Atrophic Long-Bone Non-Union

**Figure 2:**
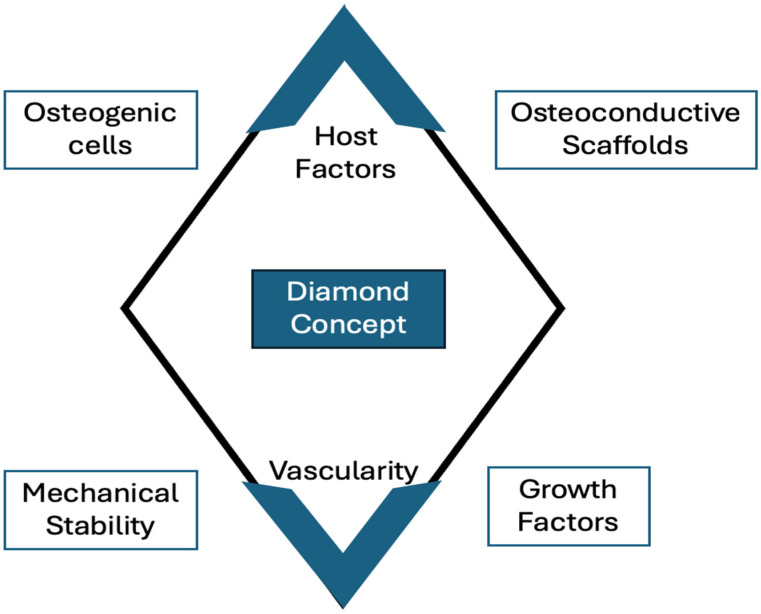
Illustration of the “diamond concept” for bone healing.

The “diamond concept” has been recently introduced for the successful management of long-bone non-union [108]. This concept highlights the important constituents that should be present for successful healing at the non-union site, including signals (osteoinductive molecules/growth factors), osteoprogenitor cells, an osteoconductive matrix (scaffold), a healthy vascular bed (promoting angiogenesis), and an optimum fracture fixation (mechanical environment) (Figure 2). The combination of signals, cells, matrix, and vascularity needs to be taken into account by the surgeon at the time of revision surgery.

**Figure 3:**
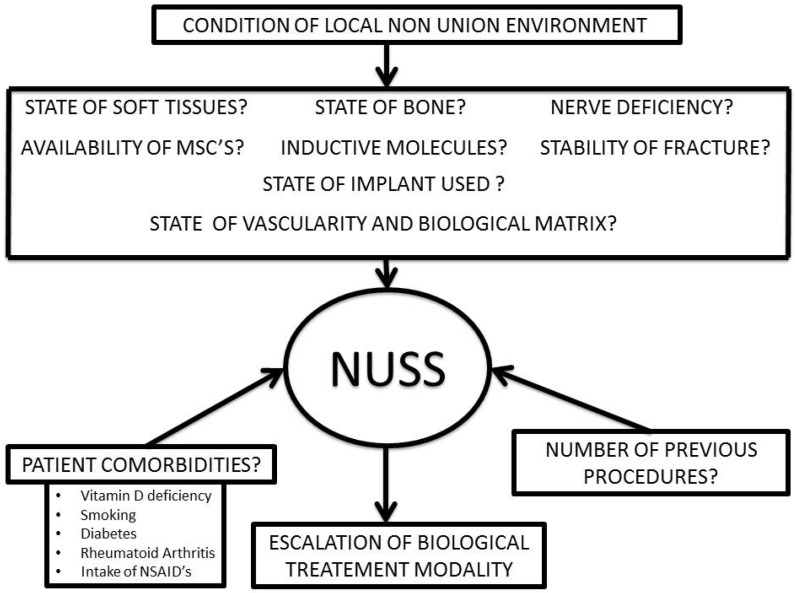
Several parameters need to be evaluated for computation of the NUSS (Non-Union Scoring System) to select the type of escalation of treatment appropriate to optimize the healing outcome of the non-union.

**Figure 4:**
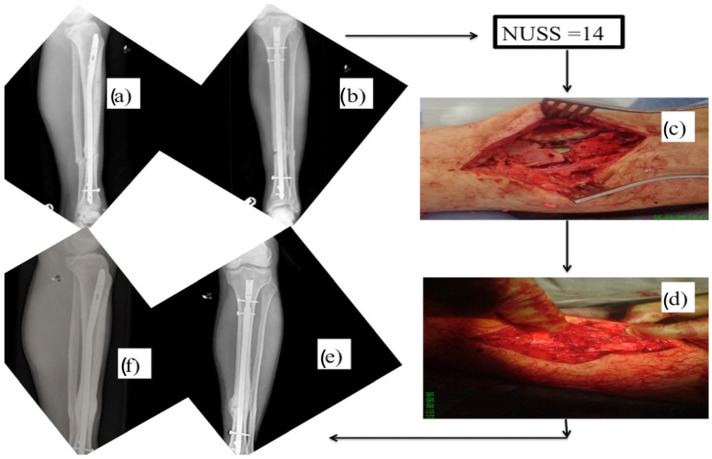
A 46-year-old male sustained left tibial fracture stabilized with an intramedullary nail. Nine months later, radiographs illustrated an atrophic tibial non-union: (**a**) lateral and (**b**) anterior–posterior view. NUSS calculated 14 points (standard treatment). As the mechanical environment was stable, bone grafting was performed only from iliac crest. (**c**) Intraoperative picture illustrating the non-union environment. (**d**) Bone graft was placed in the area of non-union. (**e**) Lateral view and (**f**) anterior–posterior view of radiographs illustrating union 3.8 months later.

The all-inclusive application of the “diamond concept” in recalcitrant cases has been reported with promising results in managing atrophic non-unions (Table 1) [49,109,110,111,112,113,114,115,116,117,118,119,120,121]. However, not all non-unions are thought to require this treatment approach, so the key issue remains how to identify cases that would benefit from this strategy. To address this issue, a comprehensive computation scoring system (Non-Union Scoring System, NUSS) has been developed to characterize inherent difficulties that have to be overcome, and an escalation-of-treatment approach has been suggested to foresee a successful healing outcome (Figure 3 and Figure 4). The NUSS score, developed in 2008 by Calori et al., assigns non-unions a composite score based on 15 different clinical and biological parameters [122]. Total scores stratify non-unions into four groups (0–25, 26–50, 51–75, and 76–100), each corresponding to recommended treatment strategies and escalating levels of intervention. Lower scores indicate a lower-risk non-union, often responding to optimization of mechanical stability and the use of non-invasive or minimally invasive adjuncts. As the scores increase, cases often require more extensive intervention and staged or combined approaches. The highest group raises concern regarding the feasibility of limb or joint salvage and may prompt consideration of amputation, arthrodesis, or prosthetic reconstruction. This scoring system has recently been validated, with studies by Calori et al., and Abumunaser and Al-Sayya demonstrating its utility as a guideline for treatment of lower extremity non-union [123,124].

Many of the more involved atrophic non-union reconstructions involve the addition of bone graft, with autograft considered to address multiple factors in the diamond concept, including osteogenic cells, growth factors, and an osteoconductive scaffold [125]. The limited available volume of autologous bone grafting and the morbidity associated with the harvesting process led to the need for development of other graft materials for biological stimulation, including allograft, xenograft, and synthetics. Recently, the concept of composite grafting (combining different graft materials prior to implantation) has been suggested where limited availability of autologous graft is present [125]. It has been suggested that selection of the appropriate graft material or the application of a composite graft depends on the chronicity of the non-union, the previous number of unsuccessful attempts, patient-associated comorbidities, and cost [125].

### 4.5. An Algorithmic Approach to the Management of Atrophic Long-Bone Non-Union

**Figure 5:**
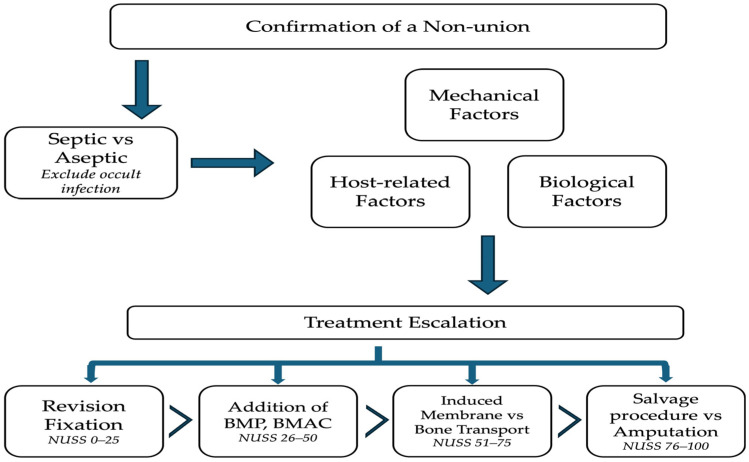
Flowchart of an algorithmic approach to management of an atrophic non-union.

Management of atrophic non-union requires an individualized, systematic approach, as demonstrated in Figure 5. The definition of a non-union varies, however. It typically is defined as a fracture that has not healed within 6–9 months and shows no evidence of progression of healing; atrophic versus hypertrophic non-unions are determined by radiographic analysis. The initial step in management is to exclude occult infection. This typically occurs through thorough clinical and laboratory evaluation. A bone biopsy may be performed when there remains a concern for occult infection after clinical and laboratory evaluation. If it is determined to be a septic non-union, an adequate debridement and confirmed eradication of infection must be performed before proceeding to additional steps focused on bony union. Once an aseptic non-union has been confirmed, the mechanical, biological, and host factors must be closely evaluated. The mechanical environment—including the alignment, stability, and appropriate use of implant, and interfragmentary strain—and any signs of implant failure must be scrutinized. The biological environment encompasses both the injury state, including degree of initial soft tissue stripping and presumed vascularity of the fracture, as well as the current bony and soft tissue defect, and soft tissue envelope. Finally, host factors should be considered. A clinical history should identify and address modifiable host factors, including poor nutrition, tobacco use, vitamin D deficiency, suboptimal glycemic control, underlying inflammatory conditions, and the use of high-risk medications.

The ultimate treatment strategy should take all of these factors into account. Although the biological activity of atrophic non-union often dominates the discussion, fracture healing cannot occur in the absence of adequate mechanical stability. Each case must be evaluated in the context of patient and injury specific factors, including the extent of the initial soft tissue injury and presumed vascularity of the fracture site, defect size, clinical suspicion of infection, and host factors influencing biological capacity. Depending on the identified mechanical, biological, infectious, and host-related factors, treatment escalation may involve a range of techniques used in isolation or in combination. These include revision fixation, bone grafting, biological adjuncts such as BMPs or bone marrow aspirate concentrate (BMAC), and composite grafting. In cases with segmental bone loss, defect management strategies such as the induced membrane technique or bone transport may be required. In advanced cases with multiple high-risk factors, salvage procedures, including arthrodesis, may be considered, and in the most severe cases, discussion with the patient regarding amputation versus continued limb salvage is warranted.

The Non-Union Scoring System (NUSS) provides surgeons with a framework to guide management and decide when to escalate to techniques such as induced membrane or staged reconstruction. Lower scores (0–25) indicate that optimization of the mechanical environment alone may be sufficient, while higher scores often necessitate both mechanical and biologic augmentation. Intermediate scores (26–50) support consideration of adjunct therapies, including non-invasive modalities (LIPUS and PEMF), or minimally invasive biologic augmentation with osteogenic cells (e.g., bone marrow aspirate concentrate, BMAC) and growth factors [122,124].

As scores increase, indicating higher-risk non-unions, more complex interventions are often required. Scores of 51–75 typically necessitate management of a defect through approaches such as the staged induced membrane technique, bone transport, or autografting, in conjunction with biologic augmentation using growth factors (BMP), osteoconductive scaffolds, and BMAC. The highest category necessitates a comprehensive evaluation of the feasibility of non-union reconstruction and consideration of joint- or limb-sacrificing procedures [122,124].

## 5. Conclusions

In summary, atrophic non-union continues to be a common complication of fracture fixation. The exact mechanical and molecular processes that lead to atrophic long-bone non-union remain obscure, and its management remain challenging and represent a significant economic burden to healthcare systems. Planned interventions to reverse this process should be well-timed and well-aimed to restore patient-related comorbidities and biological and mechanical deficiencies.

## Figures and Tables

**Table 1:** Cases of non-union treated with the “diamond concept”.

Author (Year)	Study Design	Number of Cases (Location)	Type of Non-Union	Complication Rate	Definition of Union	Length of Follow-Up	Union Rate
Wang (2025) [110]	Retrospective case–control	39 (femur)	Aseptic (atrophic)	24% (non-union, chronic pain, wound drainage)	3+ bridging cortices on XR	Minimum 12 months	100% (+PRP) 87% (−PRP)
Lu (2024) [111]	Retrospective cohort	15 (tibia)	NR	NR	RUST ≥ 10 and no further intervention	18 months	93%
Findeisen (2023) [112]	Retrospective matched-pair analysis	78 (femur, tibia)	Septic (48.7%) and Aseptic (51.3%)	NR	3+ bridging cortices on XR or CT	Minimum 12 months or full consolidation	66.7% (bone defect < 5 cm) 53.8% (bone defect ≥ 5 cm)
Chamseddine (2022) [113]	Retrospective review	53 (multiple sites)	Aseptic	7.5% (at site of ICBG)	3+ bridging cortices on XR and painless fracture site on palpation and weight bearing.	5–81 months (mean: 40.8 months)	98.1%
Tanner (2021) [114]	Retrospective case–control	202 (lower limb)	Aseptic (79.2%), Septic (20.8%)	NR	3+ bridging cortices on XR or CT	Minimum 12 months	79.4% (Aseptic) 71.4% (Septic)
Raven (2019) [115]	Prospective cohort	150 (multiple sites)	Aseptic (64%), Septic (36%)	37.3% (persistent non-union, disordered wound healing, etc.)	NR	12 months	84.4% (Aseptic) 72.2% (Septic)
Haubruck (2018) [49]	Retrospective case–control	156 (femur, tibia)	Aseptic (65.4%) Septic (34.6%)	NR	3+ bridging cortices on XR or CT	Minimum 12 months	91% (BMP-2) 58% (BMP-7) 67.9% (overall)
Moghaddam (2017) [116]	Retrospective cohort	88 (femur)	Aseptic (82%) Septic (18%)	38% (persistent non-union, material failure, etc.)	3+ planes of consolidation, no implant loosening	Minimum 12 months	95% (One-step—Aseptic, small defect) 64% (Two-step—Large defect and/or signs of infection)
Miska (2016) [120]	NR	50 (humerus)		16% (implant breakage, plate cut-out, hematoma, re-infection)	3+ bridging cortices on XR or CT	Median 12 months (11–29 months)	80.4%
Giannoudis (2015) [119]	Retrospective case series	64 (multiple sites)	Aseptic	7.8% (superficial infection, heterotopic bone, DVT)	3+ bridging cortices on AP and lateral XRs	Minimum 12 months (12–32 months)	98.4%
Moghaddam (2015) [117]	Retrospective cohort	99 (tibia)	Aseptic (65%) Septic (35%)	34% (non-union, disordered wound healing, etc.)	3+ bridging cortices, no secondary implant loosening	Minimum 12 months	84% (one-step: aseptic, defect < 1 cm) 80% (two-step—signs of infection, or defect > 1 cm)
Ollivier (2015) [118]	Retrospective case series	20 (tibia)	Aseptic	10% (infection, persistent pain)	Painless full weightbearing, bridging callous on two cortices (XR) and confirmed by CT scan.	Minimum 12 months, mean 14 months	90%
Giannoudis (2013) [109]	Retrospective case series	14 (subtrochanteric femur)	Aseptic	43% (medical complications (MI, unrelated death), DVT, PE, breakage of blade plate)	NR	Mean 26 months (16–48)	100%
Calori (2013) [121]	Retrospective cohort	19 (forearm)	Aseptic	20% donor site (infection, prolonged pain)	3+ bridging cortices on XR or CT	Minimum 12 months	89.47%

NR, not reported.

## Data Availability

No new data were created or analyzed in this study.

## Abbreviations

The following abbreviations are used in this manuscript:

MSCs	Mesenchymal stem cells
BMP	Bone morphogenetic protein
NUSS	Non-Union Scoring System
VEGF	Vascular endothelial growth factor
PDGF	Platelet-derived growth factor
SDF-1	Stromal-derived factor-1
